# Establishment and Validation of a Rapid ERA Detection Method for *Vibrio parahaemolyticus* in Exported Aquatic Products

**DOI:** 10.3390/bios16030176

**Published:** 2026-03-21

**Authors:** Ying Liang, Jiahua Wang, Yufeng Wang, Feng Xue

**Affiliations:** 1 MOE Joint International Research Laboratory of Animal Health and Food Safety, College of Veterinary Medicine, Nanjing Agricultural University, Nanjing 210095, China; 2 College of Life Sciences, China Jiliang University, Hangzhou 310018, China; 3 Sanya Institute of Nanjing Agricultural University, Sanya 572025, China

**Keywords:** *Vibrio parahaemolyticus*, Enzymatic Recombinase Amplification (ERA), aquatic product detection, rapid screening, specificity, sensitivity, on-site screening

## Abstract

To address the issues of operational complexity, long duration association, and reliance on specialized equipment with existing detection methods for *Vibrio parahaemolyticus*, this study established a rapid detection method for *V. parahaemolyticus* in exported aquatic products based on the domestically developed Enzymatic Recombinase Amplification (ERA) technology. To target the thermolabile hemolysin gene (*tlh*) and the iron-regulated virulence regulatory protein gene (*irgB*) of *V. parahaemolyticus*, highly specific ERA primers and probes were designed and screened. Two detection platforms, a colorimetric method and a fluorescent method, were developed. Method validation results showed that this detection system achieved specific amplification for all 30 tested *V. parahaemolyticus* strains, with no cross-reactivity observed with 30 other common foodborne pathogenic bacteria. The detection sensitivity for both the fluorescent and colorimetric methods reached 10^−1^ ng/μL, with a minimum detection limit of 10 CFU/25 g for artificially contaminated samples. The entire detection process, including sample preparation, requires only approximately 20 min—significantly faster than traditional culture (24–72 h) or even conventional PCR methods. Collaborative validation across five independent laboratories confirmed excellent reproducibility, with inter-laboratory agreement yielding a Kappa coefficient of 0.98. The ERA method operates at a low, constant temperature (37–39 °C), eliminating the need for thermal cyclers. When combined with portable isothermal amplification devices and visual (colorimetric) readout, it offers a distinct advantage in terms of speed, cost-effectiveness, and suitability for resource-limited or field settings compared to existing PCR-based or culture-based platforms. This method is simple to operate, rapid, sensitive, and highly suitable for on-site application, providing a reliable and practical technical solution for the rapid screening and risk monitoring of *V. parahaemolyticus* in exported aquatic products.

## 1. Introduction

*Vibrio parahaemolyticus* is a Gram-negative, halophilic coccobacillus belonging to the genus *Vibrio* within the family *Vibrionaceae* [1]. It can be classified into 13 serogroups based on O antigen differences and is widely distributed in coastal waters, seabed sediments, and marine organisms such as fish and shellfish. This bacterium is one of the main pathogens causing foodborne illness in coastal areas. Pathogenic strains produce thermostable direct hemolysin (TDH) and TDH-related hemolysin (TRH), which possess hemolytic activity, enterotoxic effects, and lethal potential [2,3], capable of causing acute diarrhea, abdominal pain, vomiting, and other symptoms in humans. In immunocompromised individuals, severe manifestations such as systemic spasms, dehydration, and acidosis may occur, posing a significant threat to food safety and public health [4].

Currently, detection methods for *V. parahaemolyticus* mainly include microbial culture and identification, physiological and biochemical methods, molecular biology methods, and immunological detection methods [5,6]. However, these methods commonly suffer from drawbacks such as cumbersome operational steps, long detection cycles (typically 24–72 h), susceptibility to subjective factors, or dependence on complex instruments [7]. With the rapid development of international trade in aquatic products, there is an urgent need to develop rapid, accurate, sensitive, and on-site applicable technical methods to achieve timely screening and risk control of *V. parahaemolyticus*.

Enzymatic Recombinase Amplification (ERA) is a novel isothermal amplification technology independently developed in China in 2019 (PCT201910262085.3). It offers advantages such as low reaction temperature, fast amplification speed (typically within 20 min), high sensitivity, and no need for complex temperature-cycling equipment, showing promising application prospects in the field of foodborne pathogen detection [8,9]. Compared to RPA, ERA utilizes a mutated, low-temperature-adapted phage enzyme system and requires the use of a probe modified with a tetrahydrofuran (THF) residue. While the amplification time of ERA (20–30 min) is slightly longer than that of RPA (10–20 min), it offers enhanced specificity and improved resistance to sample matrix inhibition through optimized enzyme engineering and probe design. Moreover, ERA is particularly suited for real-time fluorescence detection and avoids certain patent restrictions associated with RPA, making it more applicable for quantitative analysis and point-of-care testing (POCT) in food safety and clinical diagnostics. Compared to conventional PCR, ERA eliminates the need for precise temperature cycling, simplifying instrumentation requirements. More importantly, in the context of *V. parahaemolyticus* detection, ERA holds specific advantages over other prevalent isothermal techniques like Loop-Mediated Isothermal Amplification (LAMP). While LAMP requires the design of four to six primers, increasing the complexity and potential for primer-dimer formation or non-specific amplification, ERA typically utilizes only a single pair of primers and a probe, similar to PCR. This streamlined primer design reduces the risk of off-target reactions and enhances specificity—a critical factor for accurately distinguishing *V. parahaemolyticus* from closely related *Vibrio* species commonly found in aquatic samples [10]. This study designed and optimized ERA primers and probes targeting specific genes of *V. parahaemolyticus*, establishing both colorimetric and fluorescent detection systems. Systematic validation was conducted according to GB 4789.45-2023 “National Food Safety Standard General Guidelines for Validation of Microbiological Test Methods” [11] (the official link can be found in the Supplementary Materials); the ultimate goal is to provide a validated, standardized, and equipment-simplified technical solution that is convenient for rapid qualitative screening and on-site monitoring of *V. parahaemolyticus*, thereby enhancing the safety control of exported aquatic products.

## 2. Materials and Methods

### 2.1. Bacterial Strains and Samples

This study utilized a total of 60 experimental strains, including 30 strains of *V. parahaemolyticus* (covering different serogroups, virulence types, and sources) and 30 strains of other common foodborne pathogens and related bacteria (including *Vibrio alginolyticus, Listeria monocytogenes, Salmonella typhimurium, Staphylococcus aureus*, etc.). These were sourced from authoritative institutions such as the American Type Culture Collection (ATCC, Manassas, VA, USA), the German Collection of Microorganisms and Cell Cultures (DSM, Braunschweig, Germany), and the China Medical Culture Collection (CMCC, Beijing, China). All strains were preserved at the Inspection and Quarantine Microbial Culture Collection Center of China (IQCC, Beijing, China).

Validation samples included: DNA from 60 strains (concentration 10 ng/μL) for exclusivity verification; *V. parahaemolyticus* ATCC 17802 DNA at a concentration of 10^2^ ng/μL for sensitivity verification; and DNA from various artificially contaminated aquatic product samples (fish, shrimp, shellfish) contaminated with *V. parahaemolyticus* ATCC 17802 at levels of 10^3^, 10^2^, 10^1^, and 10^0^ CFU/g for practical sample detection validation.

### 2.2. Main Reagents and Instruments

Reagents included: specific ERA primers and probes (synthesized by Sangon Biotech (Shanghai) Co., Ltd., Shanghai, China.), DNA extraction buffer (formula: 10 mmol/L Tris-HCl [pH 8.0], 1 mmol/L EDTA, 0.1% SDS), ERA fluorescent nucleic acid amplification kit, chromogenic agent, activator, etc., all provided by the Chinese Academy of Inspection and Quarantine (CAIQ, Beijing, China). Culture media included 3% Tryptic Soy Broth (TSB), 3% NaCl Tryptic Soy Agar (TSA) were purchased from Oxoid, Basingstoke, UK; Alkaline Peptone Water (APW), BPW, *Vibrio* chromogenic medium, and plate count agar (PCA) were purchased from Beijing Luqiao Technology Co., Ltd. (Beijing, China).

All the following instruments were used in this experiment: Pico 17 Centrifuge (Thermo Fisher Scientific, Waltham, MA, USA); BioSpec-nano UV–Vis Spectrophotometer (Shimadzu Corporation, Kyoto, Japan); Vortex Mixer (IKA Works GmbH, Staufen, Germany); Paddle Blender (Interscience, Saint-Nom-la-Bretèche, France); Autoclave (Tuttnauer Ltd., Jerusalem, Israel); DH-420 Electric Thermostatic Incubator (Memmert GmbH + Co. KG, Schwabach, Germany); Water Bath (Jintan Baita XinBao Instrument Factory, Changzhou, China); Portable Mini qPCR Instrument (Shanghai Yihui Biotechnology Co., Ltd., Shanghai, China); and Electrophoresis System and PharosFX Automated Gel Imaging System (BIO-RAD Laboratories, Inc., Hercules, CA, USA).

### 2.3. Strain Cultivation and Bacterial Suspension Preparation

Liquid culture: *V. parahaemolyticus* was cultured in 3% TSB medium, Burkholderia gladioli pathovar cocovenenans in GVC enrichment broth, and other strains in ordinary TSB medium, all under shaking culture at 37 °C ± 1 °C and 180 rpm for 16–18 h.

Plate culture: *V. parahaemolyticus* was cultured on 3% NaCl TSA medium, B. gladioli pv. cocovenenans on modified Potato Dextrose Agar (mPDA), and other strains on ordinary TSA medium at 37 °C ± 1 °C for 24–48 h.

Bacterial suspension preparation: After activation on solid media, single colonies were picked for all strains. *V. parahaemolyticus* was diluted with 3% NaCl Alkaline Peptone Water to prepare a 10^2^ CFU/mL suspension, while non-target bacteria were diluted with Buffered Peptone Water (BPW) to prepare a 10^3^ CFU/mL suspension for nucleic acid extraction. In this study, the bacterial suspension was calibrated using the ten-fold serial dilution method combined with the plate counting method. First, a certain amount of bacterial solution was diluted in a series (such as 10^−1^, 10^−2^, 10^−3^, etc.), and then the appropriate dilution degree was selected to spread the bacterial solution on the plate culture medium. After cultivation, the colony-forming units (CFU) were counted. Finally, the original bacterial solution concentration was calculated based on the number of colonies and the dilution factor, and the bacterial suspension with the target concentration was prepared.

### 2.4. Culture of Vibrio parahaemolyticus and Extraction of Genomic DNA

If it was freeze-dried bacterial powder, it was inoculated into 5 mL of 3% NaCl TSB medium and incubated in a constant temperature incubator at 37 °C for 20 h. Taking the bacterial solution, plate streaking inoculation was conducted in 3% NaCl TSA medium, followed by incubation in a constant temperature incubator at 37 °C for 20 h. A single colony was picked and added to 5 mL of 3% NaCl TSB medium for 12 h of cultivation. Finally, 1 mL of the bacterial solution was placed in a 1.5 mL centrifuge tube.

If it was a sample to be tested, the sample preparation and enrichment culture steps were carried out according to GB 4789.7, and then 1 mL of the corresponding enrichment solution was added to a 1.5 mL sterile centrifuge tube.

The above bacterial solution was centrifuged at 12,000 *g* for 2 min, the supernatant was removed, 100 μL of DNA extraction solution was added, and the solution was vortexed and mixed well before incubating at 100 °C in a water bath for 10 min, centrifuging at 12,000 *g* for 5 min, and taking 50 μL of the supernatant as the DNA template. The concentration was then measured and the solution was stored at −20 °C.

### 2.5. Establishment of ERA Detection System

#### 2.5.1. Primer and Probe Design

Target Gene Selection: Two genes specific to *Vibrio parahaemolyticus* were selected as amplification targets: the thermolabile hemolysin gene (*tlh*), a species-specific marker, and the iron-regulated virulence protein gene (*irgB*), associated with pathogenicity. This dual-target strategy aimed to enhance the reliability and coverage of the detection method.

Primer and Probe Design: Initial candidate primers and probes were designed manually against conserved regions of the *tlh* and *irgB* genes. A total of three primer/probe sets were designed (Table 1). Set 1 (VP-1F/1R/1P) targeted the *irgB* gene. Set 2 (VP-2F/2R) and Set 3 (VP-3F/3R/3P) targeted different regions of the *tlh* gene. Notably, the primers VP-2F and VP-2R were also intended for use in the colorimetric assay. All oligonucleotides were synthesized and purified by Invitrogen (Shanghai) Trading Co., Ltd, Shanghai, China.

#### 2.5.2. Amplification System Setup

Fluorescent ERA total reaction system (50 μL): 20 μL solvent solution, 2.1 μL forward primer (10 μM, stock solution), 2.1 μL reverse primer (10 μM, stock solution), 0.6 μL probe (10 μM, stock solution), 1.0 μL DNA template, 22.2 μL sterile ddH_2_O, and 2 μL activator.

Colorimetric ERA total reaction system (50 μL): 20 μL solvent solution, 2.5 μL forward primer (10 μM, stock solution), 2.5 μL reverse primer (10 μM, stock solution), 1.0 μL DNA template, 22 μL sterile ddH_2_O, and 2 μL activator.

#### 2.5.3. Amplification Procedure and Result Interpretation

Fluorescent Method: Using a Beebird series qPCR instrument (Shanghai Yihui Biotechnology Co., Ltd. (Shanghai, China)), the reaction mixture was incubated at a constant temperature of 39 °C. After an initial 1 s step, fluorescence signals were collected at 14 s intervals for a total of 30 time points (referred to as “cycles” in the instrument software). The fluorescence signals were monitored in the FAM channel. A fluorescence threshold of 300 relative fluorescence units (RFU) was set to distinguish positive signals from background. Samples showing a distinct “S”-shaped amplification curve were retested once; a reappearing amplification curve was judged as positive, while no logarithmic fluorescence increase was judged as negative. (Note: The qPCR instrument uses the term “cycle” to denote discrete time points of fluorescence acquisition during isothermal incubation. This is distinct from thermal cycles in PCR).

To minimize the risk of cross-contamination due to aerosolized DNA, the colorimetric assay is designed to be completed within a single sealed tube. Amplification is fully terminated before the chromogenic agent is introduced. Importantly, the commercial ERA colorimetric kit utilizes specially formulated amplification reagents and chromogenic agents. The final reaction product forms a high-molecular-weight complex with the dye, which exhibits low diffusivity. Compared to traditional open-cap electrophoresis or liquid transfer steps, the risk of aerosol formation in this reaction buffer system is significantly reduced. Furthermore, the addition of the chromogenic agent requires only gentle inversion and mixing, avoiding vigorous shaking or pipetting steps, thereby further minimizing the potential for aerosol generation.

Colorimetric Method: After isothermal reaction at 37 °C for 15 min, 2 μL of chromogenic agent (SYBR Green I) was added, vortexed, and with the naked eye under day light conditions. Samples showing obvious green fluorescence were retested once; reappearing green fluorescence was judged as positive, while an orange solution was judged as negative.

### 2.6. Quality Control Standards

Each experiment included a blank control (sterile ddH_2_O replacing DNA template), a negative control (non-target bacterial DNA), and a positive control (*V. parahaemolyticus* ATCC 17802 DNA).

Fluorescent Method: The blank and negative controls should show no logarithmic fluorescence increase or amplification curve; the positive control should show a distinct “S”-shaped amplification curve. Otherwise, the experiment is invalid.

Colorimetric Method: The blank and negative controls should appear orange after adding the chromogenic agent; the positive control should show green fluorescence. Otherwise, the experiment is invalid.

### 2.7. Method Validation

Method validation was performed in strict accordance with the mandatory national standard GB 4789.45-2023 “National Food Safety Standard—General Guidelines for Validation of Microbiological Test Methods”. This standard stipulates core validation parameters for qualitative methods, including inclusivity, exclusivity (specificity), sensitivity (limit of detection, LOD), and robustness through collaborative studies. The validation workflow was designed to meet all these requirements comprehensively.

Inclusivity and exclusivity validation were conducted to evaluate the method’s ability to detect target strains and avoid cross-reactivity, respectively. A panel of 60 bacterial strains was utilized (Table 1), comprising 30 target *V. parahaemolyticus* strains (covering diverse serogroups and sources) and 30 non-target organisms. The non-target panel included closely related *Vibrio* spp. (e.g., *V. alginolyticus*, *V. vulnificus*), other major foodborne pathogens (e.g., *Salmonella* spp., *Listeria monocytogenes*, *Staphylococcus aureus*), and common environmental/flora bacteria. DNA from all strains (standardized to 10 ng/μL) was tested in parallel by the established ERA methods and the reference culture method (GB 4789.7) [12]. This design fulfills the GB 4789.45-2023 requirements for assessing both analytical sensitivity (inclusivity) and analytical specificity (exclusivity).

Sensitivity (LOD) determination followed the probabilistic (LOD_50_) model prescribed in Annex B of GB 4789.45-2023. Three representative aquatic product matrices—scallop (bivalve), cod (fish), and prawn (crustacean)—verified to be free of *V. parahaemolyticus* by the reference method, were selected as test substrates. Each matrix was artificially contaminated with *V. parahaemolyticus* ATCC 17802 at a target concentration near the expected detection limit. For each matrix, 20 test portions were analyzed alongside blank and positive controls (contaminated at 10 times the target level). The ERA methods (both fluorogenic and chromogenic) and the reference culture method were performed in parallel. The LOD_50_ and relative LOD (RLOD) were calculated using the standard Formulas (1) and (2) from GB 4789.45-2023, providing a statistical measure of method sensitivity in complex food matrices.

Collaborative validation involved five independent laboratories: Harbin Customs Technology Center, Dalian Customs Technology Center, Qingdao Customs Technology Center, Ningbo Customs Technology Center, and Xiamen Customs Technology Center. A standardized protocol, reagent set, and sample panel (including inclusivity/exclusivity DNA samples, sensitivity dilution series, and artificially contaminated samples) were distributed to each laboratory. The study was designed to evaluate the method’s inter-laboratory reproducibility, a key indicator of robustness and transferability as required by GB 4789.45-2023.

## 3. Results and Analysis

### 3.1. Detection Process and Result Analysis

**Figure 1:**
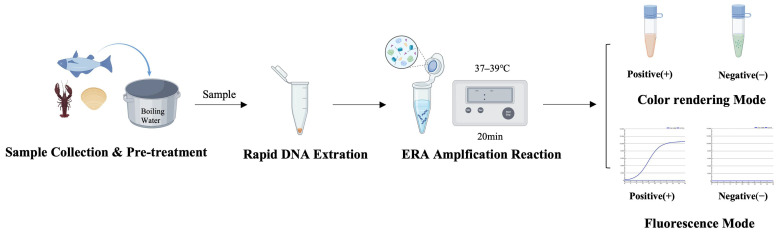
Model diagram of the detection process and result interpretation.

The rapid detection method for *Vibrio parahaemolyticus* established in this study begins with homogenization of aquatic product samples and rapid DNA extraction using a boiling method (Figure 1). Subsequently, ERA isothermal amplification is performed at a constant temperature of 37–39 °C for 20 min using primers and probes targeting the *tlh* and *irgB* genes. Results can be interpreted via a dual-mode system: in the fluorescence mode, a positive result is indicated by an “S”-shaped FAM signal curve monitored in real time with a portable instrument; in the colorimetric mode, SYBR Green I dye is added after amplification, and visible green fluorescence indicates a positive result.

### 3.2. Screening of Fluorogenic Primers and Probes

**Figure 2:**
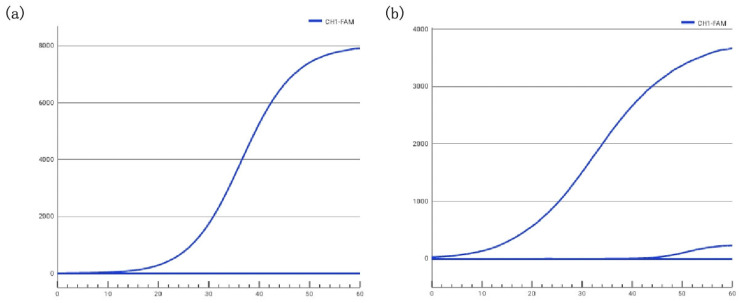
Comparison of amplification efficiency between two primer-probe sets targeting *Vibrio parahaemolyticus* ATCC 17802. (**a**) Amplification curve using the VP-1F/1R/1P set. (**b**) Amplification curve using the VP-3F/3R/3P set. The VP-1F/1R/1P set showed higher fluorescence intensity and earlier amplification compared to VP-3F/3R/3P, indicating higher amplification efficiency.

Using *Vibrio parahaemolyticus* ATCC 17802 as the template, ERA assays were performed with the primer-probe sets VP-1F/1R/1P and VP-3F/3R/3P, respectively. The detection efficiencies of the two methods were compared based on their fluorescence amplification curves. The results showed that amplification with the VP-1F/1R/1P set yielded higher fluorescence values and earlier cycle threshold (Ct) compared to the VP-3F/3R/3P set (Figure 2), indicating a higher amplification efficiency. Therefore, the VP-1F/1R/1P set was selected for further experimental analysis. This observed efficiency difference likely stems from a combination of factors inherent to the primer/probe sequences, including lower propensity for secondary structure formation and more optimal thermodynamics for the ERA enzymatic complex, which will be further explored in the discussion.

### 3.3. Analysis of Specificity and Inclusivity Using the Fluorogenic Method

**Figure 3:**
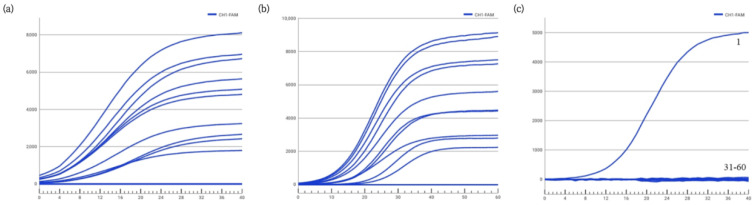
Inclusivity and specificity testing of the ERA fluorogenic assay. (**a**,**b**) Amplification curves of 30 *Vibrio parahaemolyticus* strains (inclusivity test). All strains showed positive amplification. (**c**) Amplification curves of 30 non-target bacteria (specificity test). No amplification was observed for any non-target strain, indicating high specificity.

Further analysis was conducted on the specificity and inclusivity of the VP-1F/1R/1P primer-probe set. The results indicated that the VP-1F/1R/1P combination successfully amplified all 30 strains of *Vibrio parahaemolyticus* (No. 1–30), with clear amplification curves (Figure 3a,b), and showed no amplification for 30 other common foodborne pathogenic bacteria (Figure 3c), including Bacillus subtilis and Staphylococcus aureus. The lack of cross-reactivity with non-target bacteria demonstrated high specificity. Consequently, the VP-1F/1R/1P primer-probe set was selected for establishing the subsequent rapid ERA fluorogenic detection method. Detailed strain information and results are provided in Tables S1 and S2.

### 3.4. Screening of Primers for the Chromogenic Method

**Figure 4:**
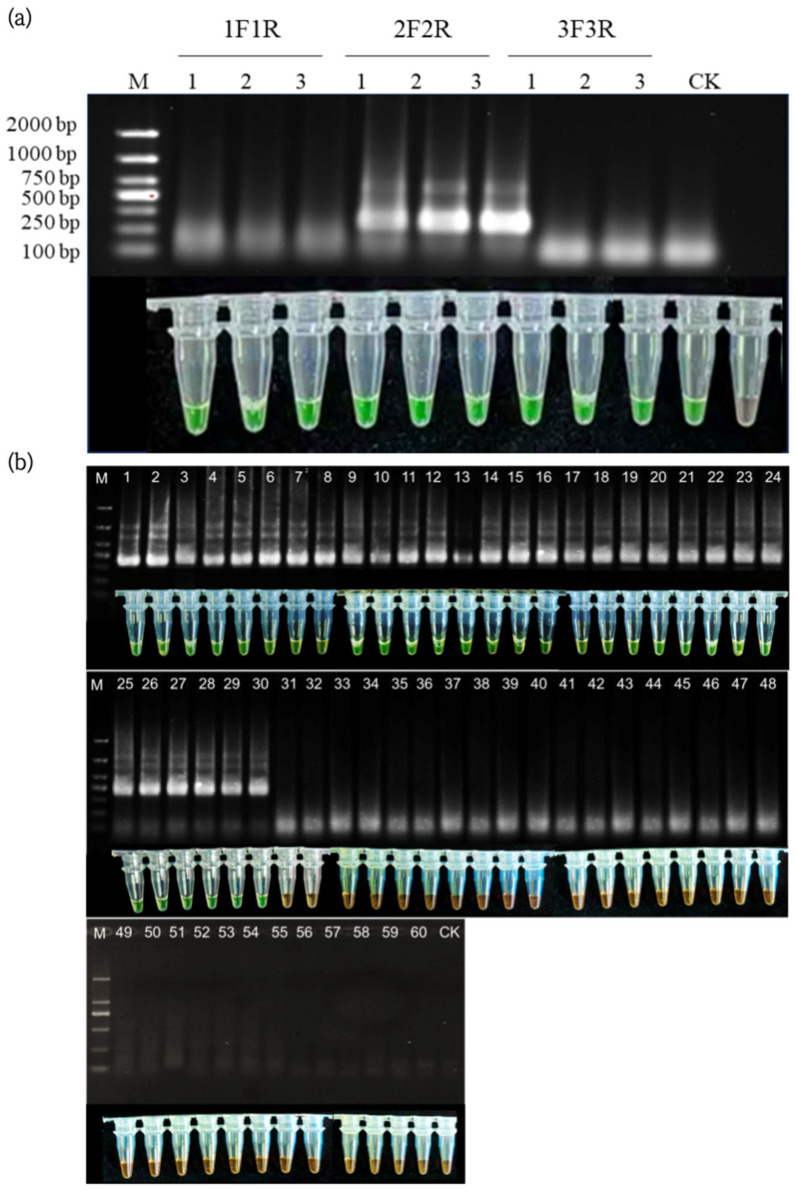
Primer screening and specificity/inclusivity testing for the ERA chromogenic assay. (**a**) Comparison of amplification efficiency among three primer sets (VP-1F/1R, VP-2F/2R, and VP-3F/3R) using three *Vibrio parahaemolyticus* strains. The VP-2F/2R set showed the highest amplification efficiency, as indicated by the brightest green fluorescence. (**b**) Test results for the specificity and inclusiveness of the ERA enzyme colorimetric method for detecting the *Vibrio* genus (*V. parahaemolyticus*). The agarose gel electrophoresis image shows the amplified products. The first to the 30th lanes: *Vibrio* strain (details see Tables S1 and S5). The 31st to the 60th lanes: non-target bacterial species (details see Tables S2 and S5). The complete list of the corresponding relationship between each lane and the specific strain number is shown in Table S5. M: 100 base pair DNA scale (key bands have been labeled). The corresponding colorimetric results are shown in the figure: green indicates a positive reaction, and orange indicates a negative reaction. (The electrophoresis (**a**) and colorimetric (**b**) results shown in this figure were both obtained using the primer pair for the parvovirus 2F/2R (target gene *tlh*)).

Three strains of *Vibrio parahaemolyticus* were used as templates to screen primers for the ERA chromogenic assay, employing the primer sets VP-1F/1R, VP-2F/2R, and VP-3F/3R, respectively. The results, as shown in Figure 4a, demonstrated that all three primer sets successfully detected the three strains of *Vibrio parahaemolyticus*, indicating good inclusivity. However, the VP-2F/2R set showed the brightest green fluorescence and the shortest time to positivity, indicating significantly higher amplification efficiency compared to the VP-1F/1R and VP-3F/3R sets. Consequently, the VP-2F/2R primer set was selected for further specificity analysis.

### 3.5. Specificity and Inclusivity Tests for the Chromogenic Method

Specificity analysis was further conducted on the previously screened primer set VP-2F/2R, which exhibited favorable amplification efficiency and inclusivity. The amplification products were analyzed using both electrophoresis and the chromogenic method. The results are presented in Figure 4b.

The results indicate that only ERA reactions using the 30 strains of *Vibrio parahaemolyticus* (No. 1–30) as templates produced amplification bands, with the chromogenic reaction turning green. In contrast, ERA reactions using other bacterial species as templates yielded no amplification bands, and the chromogenic reaction remained orange. This demonstrates that the VP-2F/2R primer set possesses excellent specificity and is suitable for use as a primer set in the detection of *Vibrio parahaemolyticus*.

### 3.6. Sensitivity Test with Actual Samples

Sensitivity validation was conducted in accordance with GB 4789.45-2023 “National Food Safety Standard—General Principles for Validation of Microbiological Examination Methods”. One sample each of bivalve mollusk (scallop), fish (cod), and shrimp (prawn), previously confirmed to be free of *Vibrio parahaemolyticus* by GB 4789.7-2013 [12], were selected as the spiking matrices. *Vibrio parahaemolyticus* ATCC 17802 was used as the quality control strain. The concentration of the spiked bacterial inoculum was set around the limit of detection (LOD_50_). For each matrix, 20 replicate tests were performed, along with one blank control and one positive control. The contamination level of the positive control was 10 times the concentration of the bacterial inoculum spiked at the LOD level. The sensitivity of the ERA fluorogenic method, the ERA chromogenic method established in this study, and the traditional culture method was analyzed. The LOD_50_ and RLOD were calculated using Formula (1) and Formula (2), respectively, as follows:

#### Calculation of LOD_50_

The LOD_50_ was calculated according to Formula (1), with the unit expressed as “CFU”.

(1) $${LOD}_{50}=\frac{0.7\times d\times m}{\ln[n/(n-y)]}$$

In the formula:

d—the acceptable reference value for the sample, expressed in CFU/g (mL);

m—the test portion amount, expressed in g (mL);

n—the total number of tests;

y—the number of positive results.

The RLOD was calculated according to Formula (2).

(2) $$ROLD=\frac{\ln[n_{\mathrm{ref}}/(n_{\mathrm{ref}}-y_{\mathrm{ref}})]}{\ln[n_{\mathrm{val}}/(n_{\mathrm{val}}-y_{\mathrm{val}})]}$$

In the formula:

n_ref_—the total number of tests for the reference method;

y_ref_—the number of positive results for the reference method;

n_val_—the total number of tests for the method to be validated;

y_val_—the number of positive results for the method to be validated.

**Figure 5:**
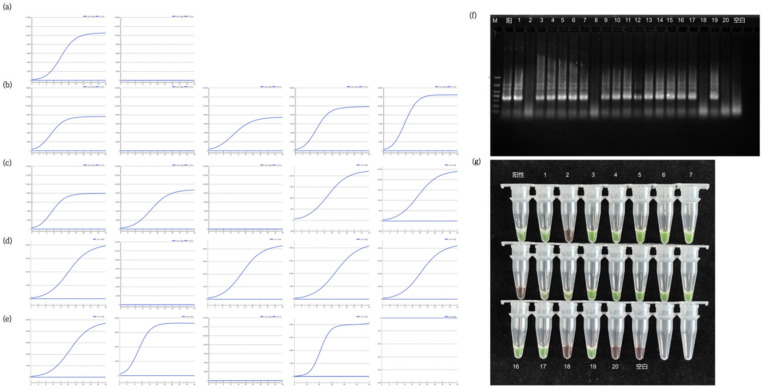
(**a**–**e**) Sensitivity results of the ERA fluorogenic method for detecting *Vibrio parahaemolyticus* (cod). In (**a**), the leftmost is the positive sample (green fluorescence indicates amplification) and the rightmost is the blank (no fluorescence); (**b**–**e**) show the results of 20 cod samples (green fluorescence indicates a positive detection, while absence of fluorescence indicates a negative result); (**f**,**g**) sensitivity results of the ERA chromogenic method for detecting *Vibrio parahaemolyticus* (cod) (Green color indicates a positive reaction, and orange color indicates a negative reaction).

**Figure 6:**
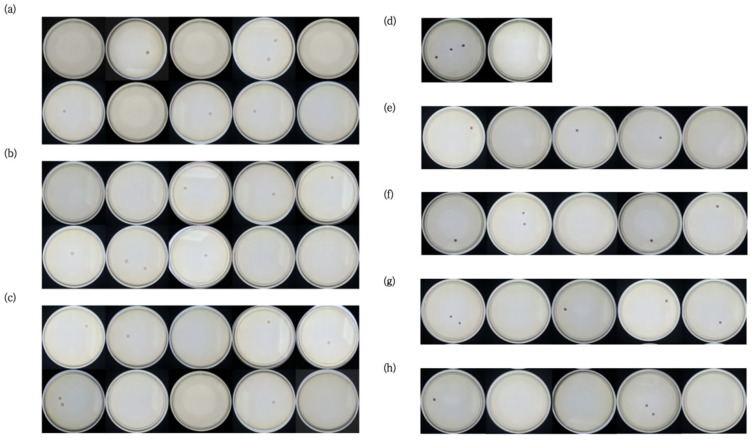
(**a**–**c**) Plate count results of bacterial suspensions (showing the plate count results of *Vibrio parahaemolyticus* suspensions in the three aquatic product matrices: scallop, cod, and prawn, respectively); (**d**–**h**) detection results of *Vibrio parahaemolyticus* by the traditional culture method (cod) ((**d**): the leftmost is a positive sample, the rightmost is a negative sample; (**e**–**h**) represent the 20 test samples for cod).

The results showed that the outcomes of the ERA fluorogenic method, the ERA chromogenic method, and the traditional culture method for *Vibrio parahaemolyticus* in the three aquatic product matrices (scallop, cod, and prawn) were consistent. The LOD_50_ was calculated, confirming that the method’s sensitivity (LOD_50_) reached 0.35~0.53 CFU, and the RLOD ranged from 0.87 to 1. The sensitivity analysis results for each matrix are presented in Table S4, and partial matrix sensitivity analysis results are shown in Figure 5 and Figure 6.

In this study, The LOD_50_ (0.35–0.53 CFU) is a statistical parameter derived from the binary outcome data of the low-level spiking experiment, reflecting the microbial count at which the probability of detection is 50% under these specific experimental conditions. For the detection of actual samples, following 1:10 homogenization and 1 mL sampling for testing, the minimum stable limit of detection of this method in three aquatic product matrices was 10 CFU/25 g (Table 2). This result was consistent with the LOD_50_ value in terms of the order of magnitude and had been verified using artificially contaminated samples, which demonstrates that the method possesses high sensitivity and reliability in practical applications.

### 3.7. Detection of Artificially Contaminated Samples

Fish, shrimp, and shellfish samples were artificially contaminated at different levels (10^3^, 10^2^, 10^1^, 10^0^ CFU/25 g). After sample pretreatment and DNA extraction, detection was performed. Results showed that the method could stably detect samples artificially contaminated with 10 CFU/25 g in all three types of aquatic products, with a detection rate of 100%. Samples contaminated with 10^0^ CFU/25 g were not detected. This indicates that the minimum detection limit of this method in actual food matrices is 10 CFU/25 g, meeting the requirements for rapid screening of *V. parahaemolyticus* contamination in aquatic products (Table 2).

### 3.8. Testing of Commercial Samples

Of the commercial aquatic samples purchased, 29 were sampled, homogenized, and inoculated for 12 h according to GB 4789.7-2013. Then, genomic DNA was extracted. The detection was conducted using the methods specified in GB 4789.7-2013 and the ERA colorimetric method and fluorescence method established in this study. The detection effect of the ERA method established was analyzed based on the test results. During the experiment, sterile ddH_2_O was used as a blank control, and the experiment was repeated three times. The results are shown in Table 3. Only one sample from the shellfish group was found to contain *Vibrio parahaemolyticus*, with an incidence rate of 3.45%. The test results were consistent with those of GB 4789.7-2013. This confirmed that the two rapid ERA detection methods established in this study are equivalent to GB 4789.7-2013 in terms of accuracy and can achieve accurate and rapid detection.

### 3.9. Collaborative Verification Results

Five verification units conducted the validation according to a unified experimental protocol. Results showed high consistency in inclusivity, exclusivity, sensitivity, and artificially contaminated sample detection results, with no significant differences (Kappa coefficient = 0.98), further confirming the stability and reliability of the method. The entire detection process (including DNA extraction) required only 20 min, significantly shortening the detection cycle compared to traditional culture methods (24–72 h) and conventional PCR (1–2 h). Moreover, it does not require large, sophisticated instruments, enabling on-site rapid detection [13].

The sample kit employed for collaborative verification comprised 60 DNA samples (30 strains of *V. parahaemolyticus* and 30 strains of non-target bacteria), along with serially diluted target DNA and artificially contaminated samples. All participating verification institutions performed the detection using unified reagents, instruments and standard operating procedures (SOPs). The overall results demonstrated that for the qualitative exclusion test panel containing 30 positive and 30 negative samples, the detection results from the five laboratories were in complete accordance with the expected outcomes, with no false positive or false negative reports recorded. Consequently, the calculated overall Kappa coefficient was 0.98, indicating an almost perfect agreement. For the sensitivity tests, all laboratories reported that the limit of detection (LOD) of the fluorescent method and chromogenic method for pure DNA was 10^−1^ ng/μL, and both methods could stably detect *V. parahaemolyticus* at the level of 10 CFU per 25 g in artificially contaminated samples. Throughout the entire verification process, no inter-laboratory inconsistent results requiring arbitration were observed.

## 4. Discussion

Rapid and accurate detection of *V. parahaemolyticus* is a key link in ensuring the quality and safety of aquatic products. Traditional microbial culture methods require multiple steps such as enrichment, isolation, purification, and biochemical identification, resulting in long detection cycles that are difficult to meet with regard to the rapid detection needs of scenarios like port clearance and enterprise quality control [14]. Molecular biology methods like real-time fluorescent PCR, while relatively sensitive, depend on precision equipment like PCR instruments and have issues such as relatively long amplification times and high operational requirements [15]. Other isothermal amplification technologies like LAMP, which requires the design of four primers, are prone to primer-dimer formation [16] and their specificity and stability need improvement.

ERA technology, as a novel isothermal amplification technology independently developed in China, is increasingly used in the field of foodborne pathogen detection due to its advantages of speed [17], sensitivity, and operational simplicity. The ERA detection method established in this study targets the *tlh* and *irgB* genes specific to *V. parahaemolyticus*. The *tlh* gene is a species-specific gene present in all *V. parahaemolyticus* strains [18,19], and the *irgB* gene is a virulence-related gene, which enhances the targeting of the detection [20,21]. By optimizing primer and probe design, the detection specificity was further improved, effectively avoiding cross-reactions with other common pathogens.

This method employs isothermal amplification at 37–39 °C, requires no temperature-cycling equipment, and can be completed using a portable amplifier, making it suitable for on-site rapid screening [22]. Furthermore, the establishment of two detection modes meets the needs of different scenarios: the fluorescent method is suitable for quantitative analysis and batch detection, providing objective and accurate results [23]; the colorimetric method is simpler to operate, allowing visual result interpretation without special instruments, making it suitable for basic-level testing institutions and on-site screening [24].

Validation results indicate that the method has a detection sensitivity of 10^−1^ ng/μL and a minimum detection limit of 10 CFU/25 g in actual samples. Compared to existing standard methods such as SN/T 1870-2016 “Detection Method for Foodborne Pathogens in Exported Food—Real-time Fluorescent PCR Method,” this method is faster and simpler to operate, while maintaining high sensitivity and specificity [25]. Furthermore, when compared to another prevalent isothermal amplification technique, Loop-Mediated Isothermal Amplification (LAMP), our ERA method offers the advantage of simpler primer design (a single pair versus 4–6 primers for LAMP), which inherently reduces the risk of non-specific amplification and primer-dimer formation, thereby enhancing assay specificity [10]. This theoretical advantage is strongly supported by our experimental data, which demonstrated no cross-reactivity with any of the 30 non-target bacterial strains tested, including closely related Vibrio species. Additionally, this method is low-cost, easy to standardize, and convenient for promotion and application in basic-level testing institutions and food enterprises. It holds significant importance for enhancing the quality and safety control level of China’s exported aquatic products and preventing foodborne disease risks.

An interesting observation during assay development was the superior amplification efficiency of the *irgB*-targeting primer/probe set (VP-1) compared to the *tlh*-targeting set (VP-3). While both genes are specific to *V. parahaemolyticus*, the underlying reasons for this difference were further explored through post hoc sequence analysis. This analysis revealed that the VP-1 set exhibited a lower predicted tendency for forming intramolecular hairpins and primer dimers compared to the VP-3 set. Such structural characteristics can significantly influence the efficiency of recombinase-mediated primer binding and strand displacement, thereby affecting the overall kinetics of the ERA reaction. This finding highlights that, beyond careful target gene selection, meticulous in silico evaluation of primer and probe secondary structures is critical for optimizing ERA assay performance—a consideration valuable for researchers developing similar isothermal amplification assays.

This study also has certain limitations, such as not validating more aquatic product matrices (e.g., cephalopods, crustaceans) [26] and not conducting large-scale clinical sample testing. Follow-up research could further optimize sample pretreatment methods, simplify DNA extraction steps, and shorten the overall detection time; expand the application validation of this method to other aquatic product matrices to improve its scope of application [27]; and combine it with CRISPR/Cas technology to further enhance detection sensitivity and specificity, developing multi-target simultaneous detection systems to provide more comprehensive technical support for food safety supervision [28].

## Figures and Tables

**Table 1:** Information on primer and probe sequences.

No.	Primer/Probe Name	Sequence (5′-3′)	Target Gene
1	VP-1F	CCATTCGCGTCGCCAAACTACATCGGAAAAC	*irgB*
	VP-1R	TGTTCTGGGTGCGAGAGTAGCAAACCATTC	
	VP-1P	CCACCCGAGAGAACTAAACAAACATCCG [FAM-dT]G[THF]A[BHQ1-dT]AGATTTTGGGTCG-C3 spacer	
2	VP-2Fa	TACTCAACACAAGAAGAGATCGACAAAA	*tlh*
	VP-2Ra	GCTACTTTCTAGCATTTTCTCTGC	
3	VP-3F	TGGTATCGCACCAGCTACTCGAAAGATGATCC	*tlh*
	VP-3R	GTGGTTGTATGAGAAGCGATTGTCAGCGGCG	
	VP-3P	AACGAAGATGGTAGCTACTTCACCATTGACGGC [FAM-dT][THF][BHQ1-dT]GGTGGAGCTCCGTT-C3 spacer	

Note: a Also used as primers for the chromogenic assay.

**Table 2:** Detection results for artificially contaminated samples (*n* = 3).

Sample Type	Contamination Level (CFU/25 g)	Fluorescent Method Positives	Colorimetric Method Positives	Detection Rate (%)
Fish	10^3^	3	3	100
Fish	10^2^	3	3	100
Fish	10^1^	3	3	100
Fish	10^0^	0	0	0
Shrimp	10^3^	3	3	100
Shrimp	10^2^	3	3	100
Shrimp	10^1^	3	3	100
Shrimp	10^0^	0	0	0
Shellfish	10^3^	3	3	100
Shellfish	10^2^	3	3	100
Shellfish	10^1^	3	3	100
Shellfish	10^0^	0	0	0

**Table 3:** Detection results of actual samples.

No.	Category	Sample	GB 4789.7-2013	ERA Fluorescent Method	ERA Chromogenic Method
1	Mollusks	Whelk	−	−	−
2	Mollusks	Oyster	−	−	−
3	Mollusks	Raw Oyster	+	+	+
4	Mollusks	Scallop	−	−	−
5	Mollusks	Mussel	−	−	−
6	Mollusks	Shortnecked Clam	−	−	−
7	Mollusks	Hard Clam	−	−	−
8	Mollusks	Corbicula Fluminea (Yellow)	−	−	−
9	Mollusks	Corbicula Fluminea (White)	−	−	−
10	Mollusks	Razor Clam	−	−	−
11	Fishes	Large Yellow Croaker	−	−	−
12	Fishes	Small Yellow Croaker	−	−	−
13	Fishes	Cod Fillet	−	−	−
14	Fishes	Hairtail	−	−	−
15	Fishes	Grass Carp	−	−	−
16	Fishes	Common Carp	−	−	−
17	Fishes	Yellow Catfish	−	−	−
18	Fishes	Atlantic Mandarin Fish	−	−	−
19	Fishes	Salmon	−	−	−
20	Fishes	Rainbow Trout	−	−	−
21	Fishes	Blotched Snakehead	−	−	−
22	Fishes	Golden Pompano	−	−	−
23	Fishes	Silver Pomfret	−	−	−
24	Fishes	Flounder Sole	−	−	−
25	Prawns	Prawns & Shrimps|White Shrimp	−	−	−
26	Prawns	Prawns & Shrimps|Macrobrachium nipponense	−	−	−
27	Prawns	Prawns & Shrimps|Mantis Shrimp	−	−	−
28	Prawns	Prawns & Shrimps|Crayfish	−	−	−
29	Prawns	Prawns & Shrimps|Red Shrimp	−	−	−

**Note:** “+” indicates positive detection result; “−” indicates negative detection result.

## Data Availability

The original contributions presented in this study are included in the article and Supplementary Material.

## Supplementary Materials

The following supporting information can be downloaded at: https://www.mdpi.com/article/10.3390/bios16030176/s1, Table S1: Strain information and experimental results of the inclusivity test for the ERA fluorogenic assay of *Vibrio parahaemolyticus*; Table S2: Strain information and experimental results of the specificity test for the ERA fluorogenic assay of *Vibrio parahaemolyticus*; Table S3: Strain information and experimental results of the inclusivity and specificity tests for the ERA chromogenic assay of *Vibrio parahaemolyticus*; Table S4: LOD_50_ detection results of the ERA fluorogenic method and the traditional culture method for *Vibrio parahaemolyticus* in three aquatic product matrices; Table S5: Correspondence between lanes in Figure 4b and the bacterial strains tested.
