# Supplementary material for: Establishment and Validation of a Rapid ERA Detection Method for Vibrio parahaemolyticus in Exported Aquatic Products
Source: Biosensors (Basel). 2026 Mar 21;16(3):176. doi: 10.3390/bios16030176 (PMC13024710; doi:10.3390/bios16030176)
Supplement: Supplementary file 1 [file biosensors-16-00176-s001.zip › biosensors-4125302-supplementary.pdf]

Table S1 Strain information and experimental results of the inclusivity test for the ERA fluorogenic assay of Vibrio parahaemolyticus

No.	Strain Name	Source and Designation	Experimental Results	
			ERA Fluorogenic Assay	GB 4789.7
1.	Vibrio parahaemolyticus	ATCC 17802	+	+
2.	Vibrio parahaemolyticus	IQCC 12312	+	+
3.	Vibrio parahaemolyticus	ATCC 33847	+	+
4.	Vibrio parahaemolyticus	IQCC 12310	+	+
5.	Vibrio parahaemolyticus	IQCC 12308	+	+
6.	Vibrio parahaemolyticus	IQCC 12309	+	+
7.	Vibrio parahaemolyticus	IQCC 12311	+	+
8.	Vibrio parahaemolyticus	IQCC 12312	+	+
9.	Vibrio parahaemolyticus	IQCC 12317	+	+
10.	Vibrio parahaemolyticus	IQCC 12322	+	+
11.	Vibrio parahaemolyticus	IQCC 12323	+	+
12.	Vibrio parahaemolyticus	IQCC 12324	+	+
13.	Vibrio parahaemolyticus	IQCC 12325	+	+
14.	Vibrio parahaemolyticus	IQCC 12326	+	+

15.	Vibrio parahaemolyticus	IQCC 12327	+	+
16.	Vibrio parahaemolyticus	IQCC 12328	+	+
17.	Vibrio parahaemolyticus	IQCC 12330	+	+
18.	Vibrio parahaemolyticus	IQCC 12331	+	+
19.	Vibrio parahaemolyticus	IQCC 12332	+	+
20.	Vibrio parahaemolyticus	IQCC 12333	+	+
21.	Vibrio parahaemolyticus	IQCC 12334	+	+
22.	Vibrio parahaemolyticus	IQCC 12335	+	+
23.	Vibrio parahaemolyticus	IQCC 12336	+	+
24.	Vibrio parahaemolyticus	IQCC 12337	+	+
25.	Vibrio parahaemolyticus	IQCC 12338	+	+
26.	Vibrio parahaemolyticus	IQCC 12339	+	+
27.	Vibrio parahaemolyticus	IQCC 12340	+	+
28.	Vibrio parahaemolyticus	IQCC 12341	+	+
29.	Vibrio parahaemolyticus	IQCC 12342	+	+
30.	Vibrio parahaemolyticus	IQCC 12343	+	+
	Blank Control (CK)		−	−

Table S2 Strain information and experimental results of the specificity test for the ERA fluorogenic assay of Vibrio parahaemolyticus

No.	Strain Name Strain	Source and Designation	Experimental Results	
			ERA Fluorogenic Assay	GB 4789.7
1.（Positive）	Vibrio parahaemolyticus	ATCC 17802	+	+
1.	Vibrio alginolyticus	ATCC 17749	−	−
2.	Vibrio fluvialis	ATCC 33809	−	−
3.	Vibrio mimicus	ATCC 33653	−	−
4.	Vibrio vulnificus	ATCC 27562	−	−
5.	Listeria monocytogenes	IQCC 22226	−	−
6.	Listeria monocytogenes 1/2a	IQCC 22291	−	−
7.	Listeria monocytogenes 1/2b	IQCC 22225	−	−
8.	Listeria monocytogenes 1/2b	IQCC 22220	−	−
9.	Listeria monocytogenes 1/2c	IQCC 22221	−	−
10.	Listeria monocytogenes 4b	IQCC 22264	−	−

11.	Listeria monocytogenes 4e	IQCC 22265	−	−
12.	EHEC O157:H7	IQCC 10195	−	−
13.	EAEC−EIEC*	IQCC 10114	−	−
14.	Bacillus cereus	IQCC 22740	−	−
15.	Bacillus cereus	IQCC 22741	−	−
16.	Pseudomonas aeruginosa	IQCC 12615	−	−
17.	β−hemolytic Streptococcus	IQCC 22106	−	−
18.	Salmonella Typhimurium	ATCC 14028	−	−
19.	Yersinia enterocolitica	IQCC 10908	−	−
20.	Yersinia enterocolitica	IQCC 10911	−	−
21.	Yersinia kristensenii	IQCC 10909	−	−
22.	Yersinia intermedia	IQCC 10913	−	−
23.	Yersinia pseudotuberculosis	IQCC 10915	−	−
24.	Shigella boydii	IQCC 11306	−	−
25.	Shigella flexneri	IQCC 10130	−	−

26.	Staphylococcus aureus	IQCC 62036	−	−
27.	Staphylococcus aureus	IQCC 22002	−	−
28.	Burkholderia gladioli pv. cocovenenans	IQCC 25108	−	−
29.	Cronobacter sakazakii	ATCC 29544	−	−
30.	Cronobacter muytjensii	ATCC 51329	−	−
	Blank Control (CK)		−	−

Table S3 Strain information and experimental results of the inclusivity and specificity tests for the ERA chromogenic assay of Vibrio parahaemolyticus

No.	Strain Name Strain	Source and Designation	Experimental Results	
			ERA Fluorogenic Assay	GB 4789.7
1.	Vibrio parahaemolyticus	ATCC 17802	+	+
2.	Vibrio parahaemolyticus	IQCC 12312	+	+
3.	Vibrio parahaemolyticus	ATCC 33847	+	+
4.	Vibrio parahaemolyticus	IQCC 12310	+	+

5.	Vibrio parahaemolyticus	IQCC 12308	+	+
6.	Vibrio parahaemolyticus	IQCC 12309	+	+
7.	Vibrio parahaemolyticus	IQCC 12311	+	+
8.	Vibrio parahaemolyticus	IQCC 12312	+	+
9.	Vibrio parahaemolyticus	IQCC 12317	+	+
10.	Vibrio parahaemolyticus	IQCC 12322	+	+
11.	Vibrio parahaemolyticus	IQCC 12323	+	+
12.	Vibrio parahaemolyticus	IQCC 12324	+	+
13.	Vibrio parahaemolyticus	IQCC 12325	+	+
14.	Vibrio parahaemolyticus	IQCC 12326	+	+
15.	Vibrio parahaemolyticus	IQCC 12327	+	+
16.	Vibrio parahaemolyticus	IQCC 12328	+	+
17.	Vibrio parahaemolyticus	IQCC 12330	+	+

18.	Vibrio parahaemolyticus	IQCC 12331	+	+
19.	Vibrio parahaemolyticus	IQCC 12332	+	+
20.	Vibrio parahaemolyticus	IQCC 12333	+	+
21.	Vibrio parahaemolyticus	IQCC 12334	+	+
22.	Vibrio parahaemolyticus	IQCC 12335	+	+
23.	Vibrio parahaemolyticus	IQCC 12336	+	+
24.	Vibrio parahaemolyticus	IQCC 12337	+	+
25.	Vibrio parahaemolyticus	IQCC 12338	+	+
26.	Vibrio parahaemolyticus	IQCC 12339	+	+
27.	Vibrio parahaemolyticus	IQCC 12340	+	+
28.	Vibrio parahaemolyticus	IQCC 12341	+	+
29.	Vibrio parahaemolyticus	IQCC 12342	+	+
30.	Vibrio parahaemolyticus	IQCC 12343	+	+

31.	Vibrio alginolyticus	ATCC 17749	–	–
32.	Vibrio fluvialis	ATCC 33809	–	–
33.	Vibrio mimicus	ATCC 33653	–	–
34.	Vibrio vulnificus	ATCC 27562	–	–
35.	Listeria monocytogenes	IQCC 22226	–	–
36.	Listeria monocytogenes 1/2a	IQCC 22291	–	–
37.	Listeria monocytogenes 1/2b	IQCC 22225	–	–
38.	Listeria monocytogenes 1/2b	IQCC 22220	–	–
39.	Listeria monocytogenes 1/2c	IQCC 22221	–	–
40.	Listeria monocytogenes 4b	IQCC 22264	–	–
41.	Listeria monocytogenes 4e	IQCC 22265	–	–
42.	EHEC O157:H7	IQCC 10195	–	–

43.	EAEC−EIEC*	IQCC 10114	−	−
44.	Bacillus cereus	IQCC 22740	−	−
45.	Bacillus cereus	IQCC 22741	−	−
46.	Pseudomonas aeruginosa	IQCC 12615	−	−
47.	β−hemolytic Streptococcus	IQCC 22106	−	−
48.	Salmonella Typhimurium	ATCC 14028	−	−
49.	Yersinia enterocolitica	IQCC 10908	−	−
50.	Yersinia enterocolitica	IQCC 10911	−	−
51.	Yersinia kristensenii	IQCC 10909	−	−
52.	Yersinia intermedia	IQCC 10913	−	−
53.	Yersinia pseudotuberculosis	IQCC 10915	−	−
54.	Shigella boydii	IQCC 11306	−	−
55.	Shigella flexneri	IQCC 10130	−	−
56.	Staphylococcus aureus	IQCC 62036	−	−

57.	Staphylococcus aureus	IQCC 22002	−	−
58.	Burkholderia gladioli pv. cocovenenans	IQCC 25108	−	−
59.	Cronobacter sakazakii	ATCC 29544	−	−
60.	Cronobacter muytjensii	ATCC 51329	−	−
	Blank Control (CK)		−	−

Table S4. LOD$_{50}$ detection results of the ERA fluorogenic method and the traditional culture method for Vibrio parahaemolyticus in three aquatic product matrices

Scallop	Experimental Results			Cod	Experimental Results			Prawn	Experimental Results		
No.	ERA Fluorogenic Assay	ERA Chromogenic Assay	Traditional Culture Method	No.	ERA Fluorogenic Assay	ERA Chromogenic Assay	Traditional Culture Method	No.	ERA Fluorogenic Assay	ERA Chromogenic Assay	Traditional Culture Method
B1	+	+	+	Y1	+	+	+	X1	+	+	−
B2	−	−	−	Y2	−	−	−	X2	+	+	+
B3	+	+	+	Y3	+	+	+	X3	+	+	+
B4	−	−	−	Y4	+	+	+	X4	−	−	−
B5	+	+	+	Y5	+	+	−	X5	+	+	+
B6	−	−	−	Y6	+	+	+	X6	−	−	−
B7	+	+	+	Y7	+	+	+	X7	+	+	+

B8	+	+	+	Y8	−	−	−	X8	+	+	+
B9	−	−	−	Y9	+	+	+	X9	−	−	−
B10	+	+	+	Y10	+	+	+	X10	+	+	+
B11	−	−	−	Y11	+	+	+	X11	−	−	−
B12	−	−	−	Y12	−	−	−	X12	+	+	+
B13	+	+	+	Y13	+	+	+	X13	+	+	+
B14	+	+	+	Y14	+	+	+	X14	−	−	−
B15	+	+	+	Y15	+	+	+	X15	+	+	+
B16	+	+	+	Y16	+	+	+	X16	+	+	+
B17	−	−	−	Y17	+	+	−	X17	−	−	−
B18	+	+	+	Y18	−	−	−	X18	−	−	−
B19	+	+	+	Y19	+	+	+	X19	+	+	+
B20	−	−	−	Y20	−	−	−	X20	+	+	+
B Positive Control	+	+	+	Y Positive Control	+	+	+	X Positive Control	+	+	+
B Negative Control	−	−	−	Y Negative Control	−	−	−	X Negative Control	−	−	−
Positive Rate	60%	60%	60%	Positive Rate	75%	75%	65%	Positive Rate	65%	65%	60%
S counting result	0.6 CFU/mL			S counting result	0.7 CFU/mL			S counting result	0.7 CFU/mL		
LOD_{50}	0.46 CFU/mL			LOD_{50}	0.35 CFU/mL			LOD_{50}	0.53 CFU/mL		
RLOD	1			RLOD	0.76			RLOD	0.87		

Table S5 Correspondence between lanes in Figure 4b and the bacterial strains tested.

Lane(s) in Fig 4b	Strain Type / Name	Source and Designation	Corresponds to Entry in
Line 1	Vibrio parahaemolyticus	ATCC 17802	Table S1 (No.1), S3
Line 2	Vibrio parahaemolyticus	IQCC 12312	Table S1 (No.2), S3
Line 3	Vibrio parahaemolyticus	ATCC 33847	Table S1 (No.3), S3
Line 4	Vibrio parahaemolyticus	IQCC 12310	Table S1 (No.4), S3
Line 5	Vibrio parahaemolyticus	IQCC 12308	Table S1 (No.5), S3
Line 6	Vibrio parahaemolyticus	IQCC 12309	Table S1 (No.6), S3
Line 7	Vibrio parahaemolyticus	IQCC 12311	Table S1 (No.7), S3
Line 8	Vibrio parahaemolyticus	IQCC 12312	Table S1 (No.8), S3
Line 9	Vibrio parahaemolyticus	IQCC 12317	Table S1 (No.9), S3
Line 10	Vibrio parahaemolyticus	IQCC 12322	Table S1 (No.10), S3
Line 11	Vibrio parahaemolyticus	IQCC 12323	Table S1 (No.11), S3
Line 12	Vibrio parahaemolyticus	IQCC 12324	Table S1 (No.12), S3
Line 13	Vibrio parahaemolyticus	IQCC 12325	Table S1 (No.13), S3
Line 14	Vibrio parahaemolyticus	IQCC 12326	Table S1 (No.14), S3
Line 15	Vibrio parahaemolyticus	IQCC 12327	Table S1 (No.15), S3
Line 16	Vibrio parahaemolyticus	IQCC 12328	Table S1 (No.16), S3
Line 17	Vibrio parahaemolyticus	IQCC 12330	Table S1 (No.17), S3
Line 18	Vibrio parahaemolyticus	IQCC 12331	Table S1 (No.18), S3
Line 19	Vibrio parahaemolyticus	IQCC 12332	Table S1 (No.19), S3
Line 20	Vibrio parahaemolyticus	IQCC 12333	Table S1 (No.20), S3
Line 21	Vibrio parahaemolyticus	IQCC 12334	Table S1 (No.21), S3
Line 22	Vibrio parahaemolyticus	IQCC 12335	Table S1 (No.22), S3
Line 23	Vibrio parahaemolyticus	IQCC 12336	Table S1 (No.23), S3
Line 24	Vibrio parahaemolyticus	IQCC 12337	Table S1 (No.24), S3

Line 25	Vibrio parahaemolyticus	IQCC 12338	Table S1 (No.25), S3
Line 26	Vibrio parahaemolyticus	IQCC 12339	Table S1 (No.26), S3
Line 27	Vibrio parahaemolyticus	IQCC 12340	Table S1 (No.27), S3
Line 28	Vibrio parahaemolyticus	IQCC 12341	Table S1 (No.28), S3
Line 29	Vibrio parahaemolyticus	IQCC 12342	Table S1 (No.29), S3
Line 30	Vibrio parahaemolyticus	IQCC 12343	Table S1 (No.30), S3
Line 31	Vibrio alginolyticus	ATCC 17749	Table S1 (No.31), S3
Line 32	Vibrio fluvialis	ATCC 33809	Table S1 (No.32), S3
Line 33	Vibrio mimicus	ATCC 33653	Table S1 (No.33), S3
Line 34	Vibrio vulnificus	ATCC 27562	Table S1 (No.34), S3
Line 35	Listeria monocytogenes	IQCC 22226	Table S1 (No.35), S3
Line 36	Listeria monocytogenes 1/2a	IQCC 22291	Table S1 (No.36), S3
Line 37	Listeria monocytogenes 1/2b	IQCC 22225	Table S1 (No.37), S3
Line 38	Listeria monocytogenes 1/2b	IQCC 22220	Table S1 (No.38), S3
Line 39	Listeria monocytogenes 1/2c	IQCC 22221	Table S1 (No.39), S3
Line 40	Listeria monocytogenes 4b	IQCC 22264	Table S1 (No.40), S3
Line 41	Listeria monocytogenes 4e	IQCC 22265	Table S1 (No.41), S3
Line 42	EHEC O157:H7	IQCC 10195	Table S1 (No.42), S3
Line 43	EAEC−EIEC*	IQCC 10114	Table S1 (No.43), S3
Line 44	Bacillus cereus	IQCC 22740	Table S1 (No.44), S3
Line 45	Bacillus cereus	IQCC 22741	Table S1 (No.45), S3
Line 46	Pseudomonas aeruginosa	IQCC 12615	Table S1 (No.46), S3
Line 47	β−hemolytic Streptococcus	IQCC 22106	Table S1 (No.47), S3
Line 48	Salmonella Typhimurium	ATCC 14028	Table S1 (No.48), S3
Line 49	Yersinia enterocolitica	IQCC 10908	Table S1 (No.49), S3
Line 50	Yersinia enterocolitica	IQCC 10911	Table S1 (No.50), S3

Line 51	Yersinia kristensenii	IQCC 10909	Table S1 (No.51), S3
Line 52	Yersinia intermedia	IQCC 10913	Table S1 (No.52), S3
Line 53	Yersinia pseudotuberculosis	IQCC 10915	Table S1 (No.53), S3
Line 54	Shigella boydii	IQCC 11306	Table S1 (No.54), S3
Line 55	Shigella flexneri	IQCC 10130	Table S1 (No.55), S3
Line 56	Staphylococcus aureus	IQCC 62036	Table S1 (No.56), S3
Line 57	Staphylococcus aureus	IQCC 22002	Table S1 (No.57), S3
Line 58	Burkholderia gladioli pv. cocovenenans	IQCC 25108	Table S1 (No.58), S3
Line 59	Cronobacter sakazakii	ATCC 29544	Table S1 (No.59), S3
Line 60	Cronobacter muytjensii	ATCC 51329	Table S1 (No.60), S3

Note: Lanes 1−30 correspond to Vibrio parahaemolyticus strains (inclusivity panel). Lanes 31−60 correspond to non−target bacterial strains (exclusivity panel). The strain information and source designation are identical to those listed in Tables S1−S3.

Link to the standard GB 4789.45−2023"National Food Safety Standard General Guidelines for Validation of Microbiological Test Methods,”

https://www.antpedia.com/standard/1747475820.html
